# Functional Nanoarchitectures For Enhanced Drug Eluting Stents

**DOI:** 10.1038/srep40291

**Published:** 2017-01-12

**Authors:** Yomna E. Saleh, Mohamed A. Gepreel, Nageh K. Allam

**Affiliations:** 1 Energy Materials Laboratory (EML), School of Sciences and Engineering, The American University in Cairo, New Cairo 11835, Egypt; 2 Department of Materials Science and Engineering, Egypt-Japan University for Science and Technology, New Borg El-Arab 21934, Alexandria, Egypt

## Abstract

Different strategies have been investigated to allow for optimum duration and conditions for endothelium healing through the enhancement of coronary stents. In this study, a nanoarchitectured system is proposed as a surface modification for drug eluting stents. Highly oriented nanotubes were vertically grown on the surface of a new Ni-free biocompatible Ti-based alloy, as a potential material for self-expandable stents. The fabricated nanotubes were self-grown from the potential stent substrate, which are also proposed to enhance endothelial proliferation while acting as drug reservoir to hinder Vascular Smooth Muscle Cells (VSMC) proliferation. Two morphologies were synthesized to investigate the effect of structure homogeneity on the intended application. The material was characterized by field-emission scanning electron microscope (FESEM), X-ray diffraction (XRD), Raman spectroscopy, energy dispersive X-ray spectroscopy (EDX), and X-ray photoelectron spectroscopy (XPS). Nanoindentation technique was used to study the mechanical properties of the fabricated material. Cytotoxicity and proliferation studies were performed and compared for the two fabricated nanoarchitectures, versus smooth untextured samples, using *in-vitro* cultured endothelial cells. Finally, the drug loading capacity was experimentally studied and further supported by computational modeling of the release profile.

Coronary heart disease (CHD) is the leading cause of death worldwide for both genders, especially in high and middle-income countries^1^,^2^,^3^,^4^. It has been intrinsically associated with atherosclerosis since the beginning of the 20^th^ century^1^. Pathologically, atherosclerosis is characterized by the formation of localized plaques within arterial walls, which hinder normal blood flow^5^. When atherosclerotic plaques are localized in one or more coronary arteries – known as coronary artery disease (CAD) – they prevent sufficient flow of oxygenated blood to the heart muscles. This results in an ischemic state at the heart muscle causing symptomatic events that range from angina pectoris to myocardial infarction, which ultimately result in sudden death^6^,^7^.

For a long time, coronary artery bypass graft (CABG) remained the gold standard practice for the treatement of coronary artery disease. However, CABG involves an invasive intervention in order to bypass blocked artery using a graft that is taken from other body parts. In 1977, the first percutaneous transluminal coronary angioplasty (PTCA) was performed to replace CABG as a minimally invasive technique, which spares CAD patients post-surgical risks and complications^1^. With plain balloon angioplasty, it was noticed that post-procedural arterial response involved 5% risk of acute restenosis during the first 24 hours, or 20–50% risk of late occlusion during the first six months, due to elastic recoiling of arterial smooth muscles. Accordingy, placement of intracoronary stents became the method of choice for PTCA to act as a residing scaffold preventing arterial collapse incurred by plain balloon angioplasty^8^. Furthermore, drug eluting stents (DES) were introduced to avoid neointimal hyperplasia caused by Bare Metal Stents (BMS). The vessel restenosis rates were decreased from 20–30% with BMS^9^ to below 10–18% with DES, revoltionaizing the field of coronary intervention. By 2006, 8 out of 10 deployed coronary stents were DES^10^, at an annual cost between 4 and 5 billion USD^11^. Clinical evaluations have so far showed strong evidence of DES superiority over BMS in reduction of in-stent restenosis rates. However, cases of serious clinical events have raised concerns on the long time safety and efficiency of DES^6^, in particular, risks of late and very late stent thrombosis^12^. Several investigations have been reported to explain why such technical marvels would turn to be thrombogenic. Some of the most supported reasons are: 1- delayed endothelialization due to locally delivered cytostatic drugs or other pathological risk factors, 2- inherent thrombogenicity of the stent as a foreign body to blood circulation, 3- hypersensitivity reactions related to the metallic material used for stent manufacturing and/or polymeric coatings used as drug carriers, 4- early discontinuation of Dual Antiplatelet Therapy (DAPT), and 5- stent malapposition or incomplete apposition, related to technical deployment^13^,^14^,^15^,^16^,^17^.

A wide range of different materials have been previously used in the manufacturing of stents. These materials need to fulfill rigorous mechanical, physical, and chemical properties, especially long term durability and immunity^18^. To this end, titanium (Ti) and its alloys have been widely used in biomedical applications, especially in dental and orthopedic applications. They show excellent biocompatibility and high corrosion resistance due to the stable oxide layer that is formed on their surfaces. However, for coronary stent manufactionring, the use of Ti has been limited to bio-inert coatings that showed significantly reduced thrombogenicity and intimal hyperplasia, such as Ti-nitride-oxide layer on Titan stent (Hexacath, France)^19^,^20^,^21^. The reason why pure titanium or some of its common alloys have not been used as stent materials is due to their high yield strength and relatively low tensile strength. Therefore, during deployment with balloon, they will need to expand to stresses greater than their yield strength. Also, with the low tensile strength and low ductility, the stent will be easily prone to fracture. In this regard, alloying Ti with other elements that would reduce its yield strength might be a good strategy to make it mechanically acceptable, while keeping its original tensile strength. Some of the promising Ti-alloys for making stents are those containing Ta and Ni^22^. Also, Ni-Ti alloys are extensively used in stents manufacturing, specially for self-expandabe stents^23^. However, Ni-hypersensitivity and toxicity have stimulated the development of Ni-free Ti-based shape memory alloys^24^.

On the other hand, coatings have been initially used to enhance the biocompatibility of stent materials within the vascular environment. Later, they were used as vehicle for drug loading and a platform to offer advanced solution for better endothelialization. However, using different materials for coating, whether polymeric or metallic, can add a layer of complexity within the manuacturing process and more importantly can be mechanically questionable during application. Also, mechanical distrbuance at the interface between the coating and the substrate can occur due to crystal mismatch. Herein, we report on nanoarchitectures that were self-grown on a newly designed Ni-free Ti-17Nb-6Ta alloy^25^ and proposed as DES surface treatment. The mecanical properties of Ti-17Nb-6Ta alloy are comparable to Nitinol, which is currently the most widely used material for self-exandable stents. Accordingly, this system is proposing a surface that is self-grown from the substrate material avoiding surface coating and crystal mismatch. The Ti-17Nb-6Ta alloy has a Young’s modulus of 68 GPa, ultimate tensile strength (UTS) of 700–1050 MPa, Elongation of 10–30% and corrosion resistance of −44.1 E_corr_ (mV). Furthermore, the tantalum content can possibly enhance the radio-opacity and stent visibility during PCTA due to its relatively high density. Two different nanomorphologies were fabricated to investigate the effect of structure homogenity on the intended application.

## Materials and Methods

### Materials

Ammonium fluoride (ACS reagent, ≥98.0%), Formamide (purum, ≥98.0% (T)) and Glycerol (ACS reagent, ≥99.5%) were purchased from Sigma Aldrich, USA. Ethylene Glycol (Pure P.A.) was purchased from Lab-Scan analytical sciences, Poland, while Ammonium Sulphate (Ex-Pure) was purchased from Oxford Laboratory, India. 2′-Deoxyadenosine drug (99%) and phosphate-buffered saline (PBS, 1X) sterile liquid were purchased from Alfa Aesar, Germany.

### Alloy fabrication

Ti-17Nb-6Ta buttons were prepared by arc-melting in high purity argon gas atmosphere. It was then homogenized at 1000 °C for 7.2 ks in same atmosphere. Subsequently, samples underwent cold-rolling by >95% thickness reduction (CR) to produce sheets of thickness 0.3 mm.

### Potentiostatic anodization

Prior to anodization, Ti-alloy sheets were cleaned ultrasonically in acetone then ethanol followed by distilled water. Platinum foil was washed in dilute HCl then distilled water. A two-electrode electrochemical cell was used for anodization with the alloy sheet as the positive electrode and Pt sheet as the negative electrode. Two different sets of conditions were used: 1- glycerol-based electrolyte containing 0.35 M NH_4_F + 5 vol% H_2_O + 20 vol% Formamide at 50 V for 2 h, and 2- aqueous-based electrolyte containing 0.11 M NH_4_F + 1 M (NH_4_)_2_So_4_ at 40 V for 2 h. After anodization, samples were ultrasonically cleaned and left to dry in air.

### Morphological characterization

The morphology of the samples was characterized using Field emission scanning electron microscope (FESEM, Leo Supra 55 – Zeiss Inc., operated at 9.00 kV). Morphological imaging was used to detect successful fabrication of nanoarchitecture, nanoindentation and cell proliferation on different morphologies. Only samples with fixed cell culture are gold-sputtered before imaging for better visuals.

### Structural and compositional characterization

Three techniques were used to identify and confirm structure and/or composition of the fabricated NTs layer on Ti-17Nb-6Ta alloy: (1) XRD diffractometer (D8, Brucker, Cu K_α_ = 1.54 Å), (2) High performance Raman Analyzer (ProRaman-L) with an excitation laser beam’s wavelength of 532 nm, (3) X-ray photoelectron spectroscopy (XPS) on a Thermo Scientific K-alpha XPS with an Al anode, with spectra charged at 532 eV reference to O 1 s.

### Mechanical characterization

Young’s modulus and hardness of the anodized samples were measured before and after annealing at 450 °C for 3 hours. Tests were done using nanoindenter XP, MTS with Berkovitch tip (20 nm) creating 6 × 6 array of indentations, separated by 150 μm. Nanoindentation was done with CSM Tip calibration mode, strain 0.05 S^−1^, depth of 3000 nm and a strain rate of 10 nm/s. FESEM was used to image the indentation projected contact area caused by the Berkovitch tip.

### Assessment of nanoarchitectures’ biological response

For biological assessment, vascular endothelial cells were extracted from mice umbilical cords and cultured *in-vitro*. No live vertebrates or human subjects were used in the experiments. The cells were used for cytotoxicity measurements, cells adhesion and proliferation on NTs. For cytotoxicity testing, MTT viability assay was conducted, using 96-well tissue culture plate with 10^4^/well. Cells were incubated at 37 °C with 5% CO_2_ in a humidified incubator for 24 hours. Absorbance was measured with microplate reader (ROBONIK TM P2000 Eliza plate reader) at 570 nm. For cell proliferation, three tests were used to study the effect of the nanoarchitecture homogeneity on tissue healing versus smooth muscle at intervals of 1, 3 and 7 days. They were furthermore used to study the effect of structure homogeneity on cells proliferation; (1) MTT viability assay mentioned earlier, (2) imaging under FESEM after gold sputtering, and (3) Trypan blue assay to count viable cells.

### Study of system capacity as drug reservoir

For drug loading, Ti-alloy sheets of 1 cm^2^ were immersed in 2′-deoxyadenosine solution (1 mg/ml) and left for 36 hours. Sheets were then removed and left to dry in air for 12 hours. Each sheet was immersed in a 10 ml beaker containing 10 ml PBS under magnetic stirring for 3 minutes at 700 rpm. Sample from the PBS solution was withdrawn and its absorbance was measured using CARY 500 UV-Vis-NIR spectrophotometer at 260 nm to calculate the drug concentration. Drug release profiles from the NTs were theoretically predicted using computational simulation. The model was built using COMSOL Multiphysics modeling software. To simulate the kinetics of drug release from the NTs, “Transport of diluted species” module was used, with Fick’s law equation governing the drug motion: (**N**_*i*_ = −**D**_*i*_ 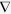
**c**_*i*,_ where for species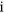, **N**_*i*_ = the molar flux (mol m^2^/s), **D**_*i*_ = the diffusion coefficient (m^2^/s), and **c**_*i*_ = the concentration (mol/m^3^)). The boundary conditions assumed that the flux outside the boundaries of the NTs and the tissue is equal to zero.

## Results and Discussion

### Morphological, Structural and Compositional Analysis of the Ti-Nb-Ta Oxide Nanotubes

**Figure 1:**
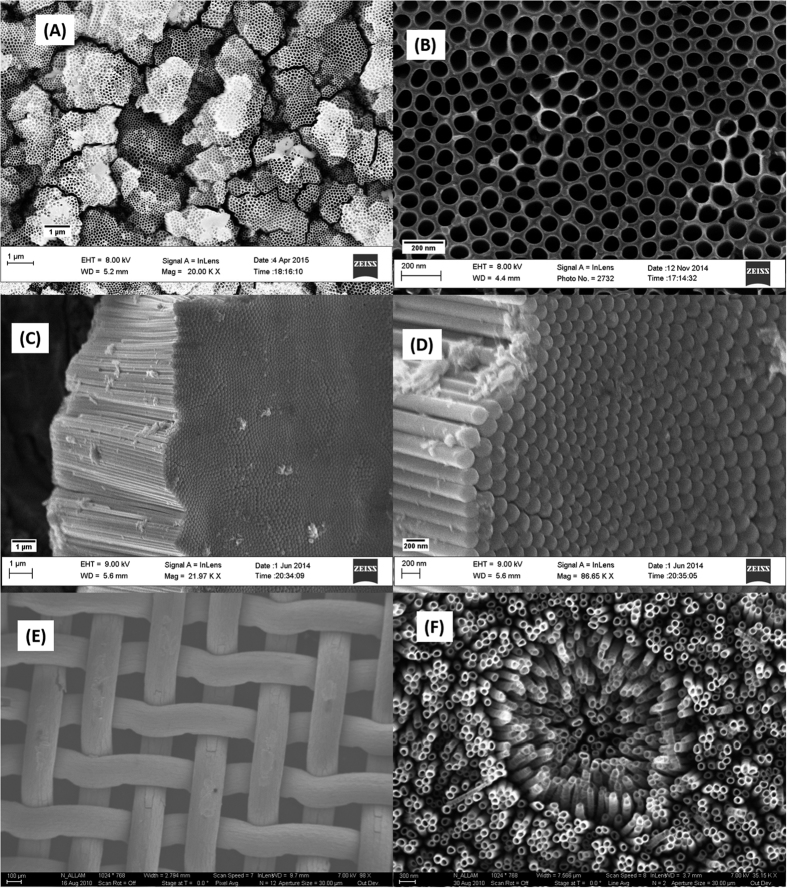
FESEM images of as-anodized Ti-17Nb-6Ta with no post thermal treatment after cold rolling. (**A**) Low magnification top-view image of the highly ordered, vertically oriented NTs, with different lengths, (**B**) high magnification top-view image of the homogenous NTs, (**C**,**D**) side views of NTs detached layers, showing NTs bottom side at low and high magnifications, respectively. (**E**,**F**) are low and high FESEM images of the nanotubes grown on uneven mesh surface.

Cold-rolled samples were directly anodized in attempt to avoid the oxide layer formation without the need for sample polishing. Upon optimizing the anodization conditions, highly ordered, vertically oriented nanotubes (NTs) were successfully self-grown on Ti-17Nb-6Ta substrate, Fig. 1. Anodization in organic electrolyte resulted in the formation of closely packed NTs with uniform diameters assembled into honey-comb-like islands separated by grooves with fused walls at the surface. The side view image of a detached layer showed highly defined NTs with distinct, uniform walls that are free from circumferential serration. Those NTs will be named *Homo-NTs* throughout the manuscript. The *Homo-NTs* have average inner diameters of 75 ± 5 nm and lengths of 12 μm. These *Homo-NTs* were studied for enhancing drug eluting stents by enhancing the material biological response and drug loading capacity as will be discussed in the following sections. Panels E and F in Fig. 1 show low and high magnification FESEM images of the NTs formed on uneven, mesh substrates.

**Figure 2:**
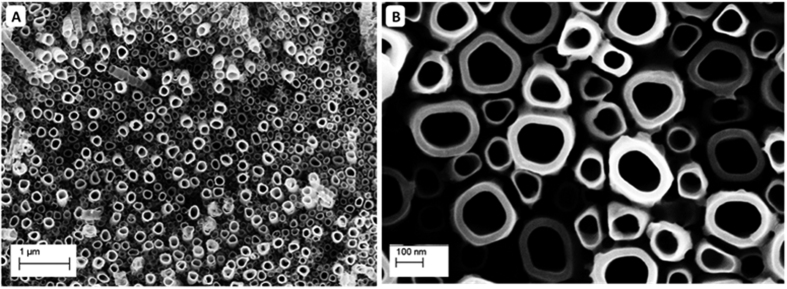
Top-view FESEM images of as-anodized Ti-17Nb-6Ta sheets in aqueous electrolyte. (**A**,**B**) show the heterogeneous NTs dimensions at low and high magnifications, respectively.

Upon anodizing the Ti-17Nb-6Ta alloy in aqueous electrolyte containing 0.11 M NH_4_F + 1 M (NH_4_)_2_SO_4_ at 40 V for 2 h, heterogeneous NTs (*Hetero-NTs*) with various inner diameters (80–190 nm) and wall thicknesses (6–28 nm) were obtained, Fig. 2. However, the length of the NTs layer was found to be ~12 μm. The effect of surface morphology (Homo *versus* Hetero- NTs) was studied for endothelial tissue biological response and system capacity to load therapeutic agents for drug eluting stents. With almost similar NTs length for the two morphologies (~12 μm), this variable was excluded from the comparison.

**Figure 3:**
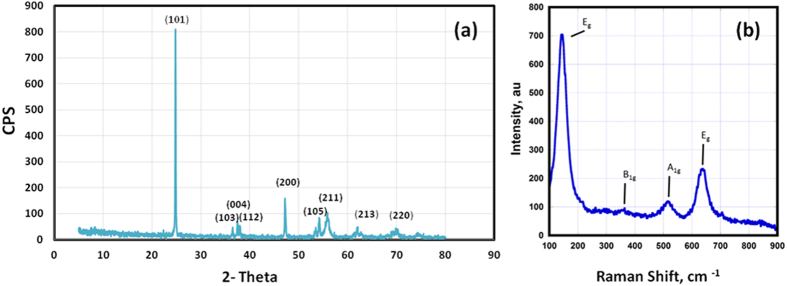
(**a**) X-ray diffraction pattern and (**b**) Raman spectra of Ti-17Nb-6Ta surface NTs annealed at 450 °C for 3 hours.

Composition and crystallinity of the fabricated Ti-17Nb-6Ta NTs samples were examined using X-ray diffraction (XRD) and Raman spectroscopy after annealing at 450 °C. Figure 3a evidently confirms the crystallization of the nanotubes in the anatase phase, with peaks corresponding to (101), (103), (004), (112), (200), (105), (211), (213) and (220) facets^26^,^27^,^28^,^29^. However, the absence or existence of the other alloy components, i.e. Nb and Ta oxides, cannot be confirmed nor denied from the XRD results. It was reported that both Nb and Ta oxides existence with Titania may not alter greatly nor postpone its phase transformation to anatase^30^,^31^,^32^. Annealed Ti-Nb, Ti-Ta-Zr and Ti-Ta surface NTs at 450 °C showed similar diffraction pattern, indicating possible overlapping of peaks from the three other components^33^,^34^,^35^.

**Figure 4:**
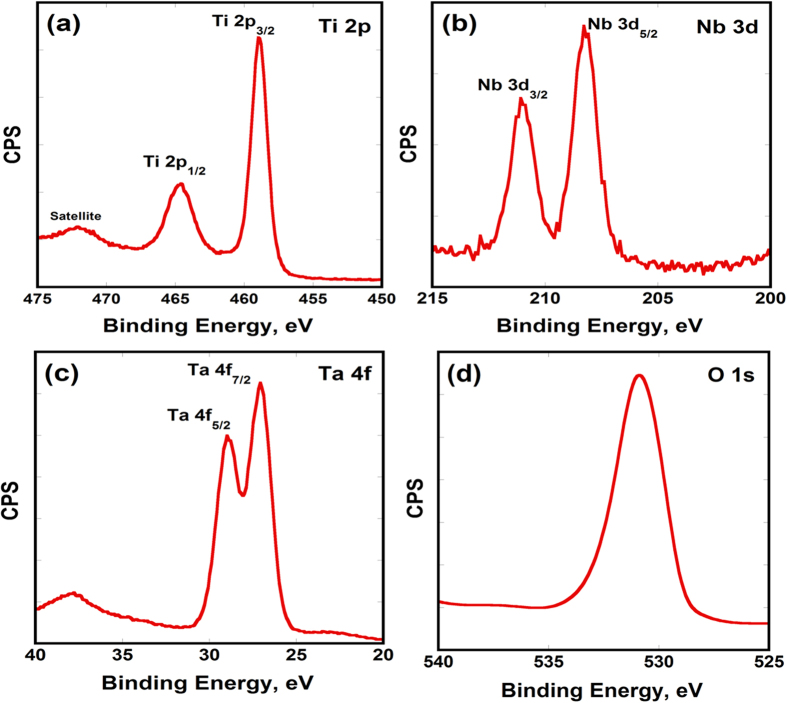
High resolution XPS spectra of (**a**) Ti 2p, (**b**) Nb 3d, (**c**) Ta 4 f and (**d**) O 1 s emissions for as-anodized Ti-17Nb-6Ta nanotubes.

Figure 3b shows the corresponding Raman spectra, indicating the tetragonal vibration mode symmetries associated with anatase; E_g_, E_g_, B_1g_ and A_1g_ modes^27^,^28^. However, slight shift and broadening of some peaks may be indicative for the presence of other elements in the crystal^36^. Yet, no distinctive peaks were revealed for Nb or Ta oxides. More sensitive and accurate technique was, therefore, needed for confirming or denying the formation of mixed oxides on the anodized surface. The three alloying elements have different activities towards etching and evidence was then needed to make sure that the three elements were retained during anodization and within the NTs. Accordingly, X-ray Photoelectron Spectroscopy (XPS) was used, which is considered a powerful tool for identifying surface components, chemical composition and oxidation state. Figure 4 shows the XPS spectra of the as-anodized Ti-17Nb-6Ta samples, showing two peaks at 464.6 eV and 458.7 eV that can be assigned to Ti 2p_1/2_ and Ti 2p_3/2_, with spin orbit splitting (Δ) of 5.9 eV associated with Ti^4+^. Panel (b) revealed Nb 3d doublet at 210.98 eV and 208.08 eV associated with Nb 3d_3/2_ and Nb 3d_5/2_, with spin orbit splitting (Δ) of 2.9 eV, confirming that signals correspond to Nb^5+^ exist. Panel (c) showed a peak at 29.08 eV for Ta 4f_5/2_ and a peak at 27.18 eV for Ta 4f_7/2_, with spin orbit splitting (Δ) of 1.9 eV, confirming the presence of Ta^5+^. Finally, panel (d) shows singlet peak at 530.98 eV corresponding to O1s, indicating the formation of metal oxide^37^.

### Nanomechanical Characterization of the Ti-Nb-Ta Oxide Nanotubes

**Figure 5:**
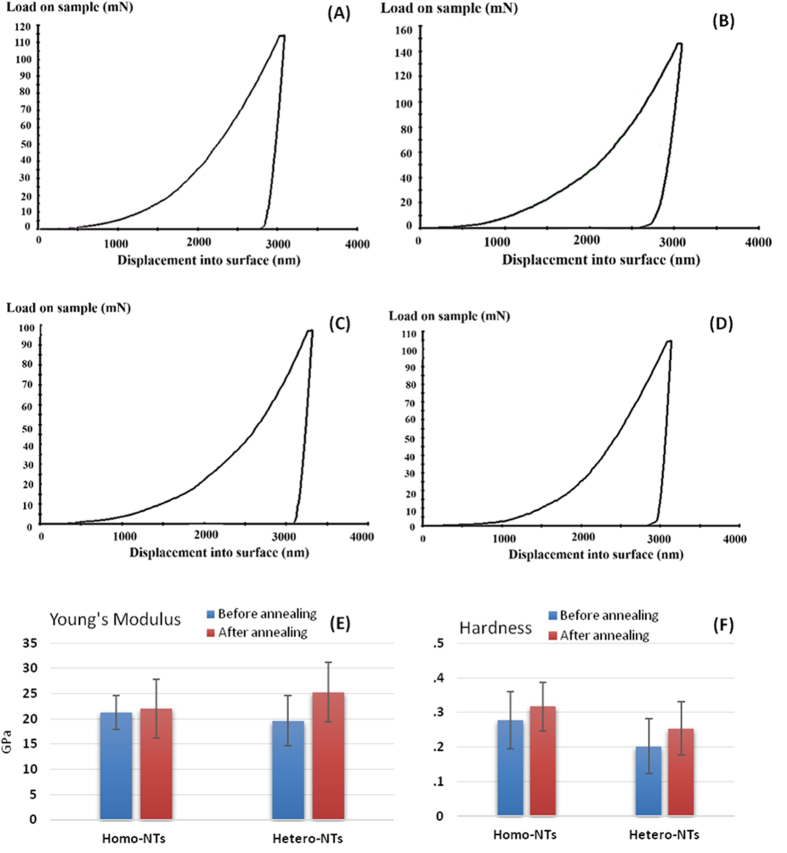
Average load-displacement curves from nanoindentation of (**A**,**B**) Homo-NTs and (**C**,**D**) Hetero-NTS, before and after annealing respectively. (**E**,**F**) Young’s modulus and hardness values for Homo- and Hetero-NTs, before and after annealing.

Not only are the stent bulk mechanical properties critical for drug eluting stents, but also the biological interaction with material surfaces is sensitive to mechanical properties at the stent/tissue interface. Surface stiffness was found to significantly influence cells fate^38^,^39^. The mechanical properties of the fabricated nanoarchitectures were investigated using the nanoindentation technique. The nanoindentation tip was used to estimate Young’s modulus (a measure of stiffness) and hardness values before and after annealing. During loading and unloading, hysteresis loop was observed, which indicated that NTs surface has elastic energy dissipation, Fig. 4. Note that the elastic recovery is higher for the annealed sample (Fig. 4(b) and (d)) compared to the as-anodized counterpart. Also, the unloading slopes of the annealed samples are found to be steeper indicating higher stiffness. The total depth of indentation is much smaller than the NTs layer thickness (12 μm). Accordingly, insights on the plastic deformation behavior of the NTs can be gained without interference from the substrate material. Young’s modulus and hardness average values were calculated and compared for Homo- and Hetero-NTs before and after annealing, Fig. 5(E) and (F) 7. It was found that both Young’s modulus and hardness increase with annealing for both Homo- and Hetero-NTs. This can be related to phase transformation upon annealing. For hardness, Homo-NTs were found to be superior. For Young’s modulus, Homo-NTs showed lower values, which do not indicate inferiority within intended application.

### Cytotoxicity and Activation of Endothelial Cells Growth

**Figure 6:**
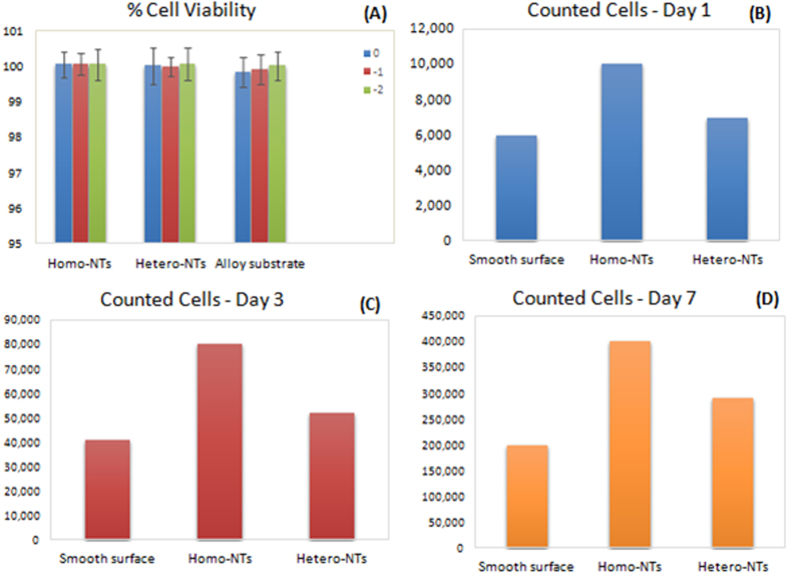
(**A**) Mean values of percentage of cell viability for 1) anodized Ti-17Nb-6Ta sheets with Homo-NTs, 2) with Hetero-NTs structures and 3) as received substrate. Illustrated for each sample, values for three different dilutions (0, −1, −2) of tested samples extracts. (**B**–**D**) Viable endothelial cells count on 1) Ti-17Nb-6Ta smooth surface 2) with Homo-NTs, 3) with Hetero-NTs structures at different time intervals (1, 3 and 7 days).

For Drug eluting stents (DES) application, material stability and cytotoxicity are considered crucial. The use of materials with any inflammatory effect can cause local tissue sensitization, which can directly affect the healing process as well as the local thrombogenicity. Figure 6A illustrates the percentage of cells survival rate or cell viability from the MTT assay for 1) Ti-17Nb-6Ta sheets with as-anodized Homo-NTs, 2) with Hetero-NTs structures, and 3) as-received substrate material. The data show three different dilutions for each sample’s extract (0, −1, −2). Note that the cells’ survival rate is extremely high for all samples at different dilutions, which excludes possible cytotoxicity from electrolyte after the sample cleaning post anodization. These results are in strong agreement with the biocompatibility and hemocomatability reports in literature for Titanium and Titanium alloys^24^. The high degree of biocompatibility is attributed to the ability of Ti-based alloys to form a stable oxide layer in most environments. Furthermore, the thicker and more stable the oxide layer, the better the material bioactivity is. This has driven earlier attempts to increase biocompatibility and activity of material surfaces by increasing the oxide layer through anodization technique^40^,^41^,^42^,^43^,^44^.

The three different samples underwent trypan blue viability assay. For each sample both dead and viable cells were counted under the microscope at specific time intervals of 1, 3 and, 7 days. Counted viable cells of the three samples are illustrated and compared, Fig. 6B–D. Note that the nanoarchitectures directed better proliferation of endothelial cells than smooth surface of the material substrate at the three studied time intervals. This indicates that surface modification of drug eluting stents with nanotopography would guide faster endothelial healing^43^. Therefore, replacing the DES polymeric coating with NTs can be of great potential towards better stenting outcome in terms of biological response. This shall not only spare the local inflammation that may be caused by polymers, but also promote endothelial tissue proper healing. Furthermore, the effect of surface homogeneity was investigated, where Homo-NTs clearly showed higher number of counted cells than Hetero-NTS, indicating better proliferation. This signifies the importance of NTs optimization in terms of surface homogeneity, which should be considered for the applications involving tissue regeneration. During endothelialization, nanoarchitectures temporarily mimic extracellular matrix (ECM), guiding and nurturing cells growth^45^,^46^. The superior outcome from Homo-NTs may be attributed to better distribution of ions, proteins and nutrients required for the growth, as well as more structured spatial guidance of the cells to grow.

**Figure 7:**
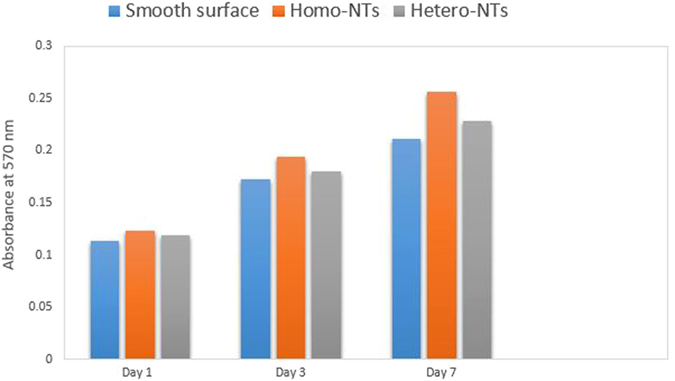
Formazan absorbance from MTT cell viability assay for: 1) Ti-17Nb-6Ta smooth surface 2) with Homo-NTs, 3) with Hetero-NTs structures at different time intervals (1, 3 and 7 days).

To confirm the trypan blue assay results, MTT assay was conducted for the same 3 types of samples. The absorbance of the formazan solutions was reported as a reflection of viable cells concentration per sample, Fig. 7. Note the better outcome upon the use of nanoarchitectured surfaces as compared to the smooth surface. Also, Homo-NTs showed the same trend reported with trypan blue assay as the superior morphology for proliferation among the three tested samples.

### FESEM Imaging of Cells Proliferation

**Figure 8:**
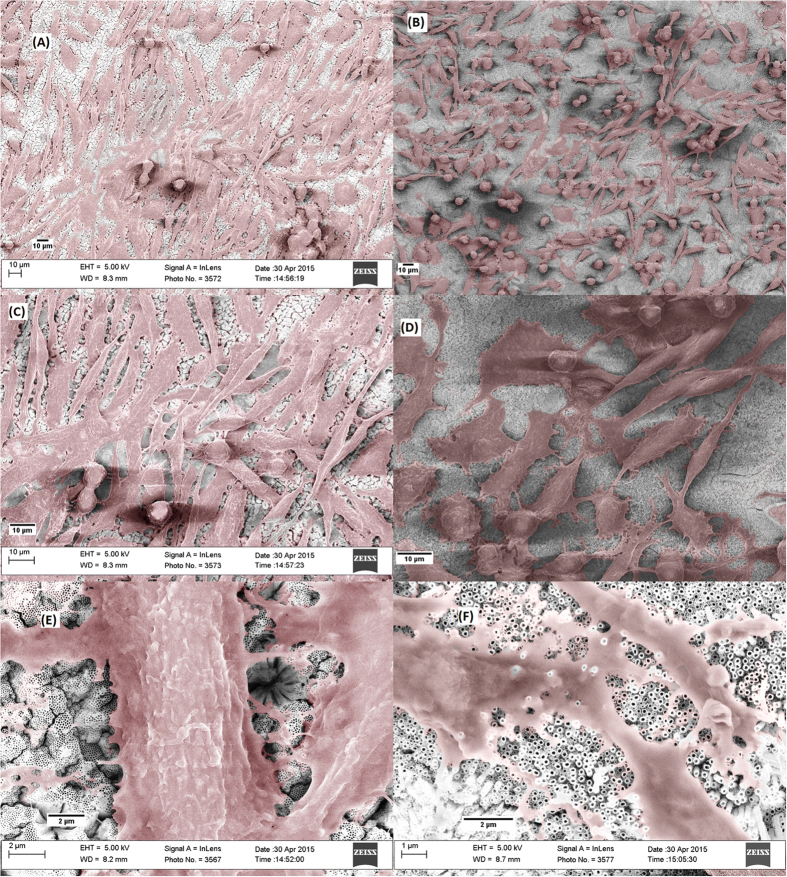
Top-view FESEM images of endothelial cells grown on NTs surfaces after 3 days. (**A**,**C**,**F**) are cells grown on Homo-NTs at different magnification. (**B**,**D**,**F**) are cells grown on Hetero-NTs at different magnification.

Endothelial cells were grown on the surface of Homo- and Hetero-NTs for 3 days to confirm the effect of surface homogeneity on the cells’ proliferation. Cells were fixed and imaged using FESEM, Fig. 8, confirming the superiority of Homo-NTs in guiding endothelialization over Hetero-NTs. Images (A),(C) and (E) of Fig. 8 represent the cells’ growth on Homo-NTs, where the island like structure of highly ordered nanotubes can be seen at the highest magnification (E). Also, images (B), (D) and (F) illustrate cells on Hetero-NTs, where at the highest magnification (F) NTs with different diameters can be observed. Elongated endothelial cells are predominantly seen on Homo-NTs, forming network-like structure with more pronounced filopodial protrusions. This indicates better cells migration and proliferation on the homogeneous NTs structure, which would directly result in higher cells count as seen in the former assays. Cells cultured on Hetero-NTs, on the other hand, showed more rounded structure, less distribution on the surface and not yet crossing into a network. This indicates potentially slower healing rates for Hetero-NTs.

### Drug loading and release

**Figure 9:**
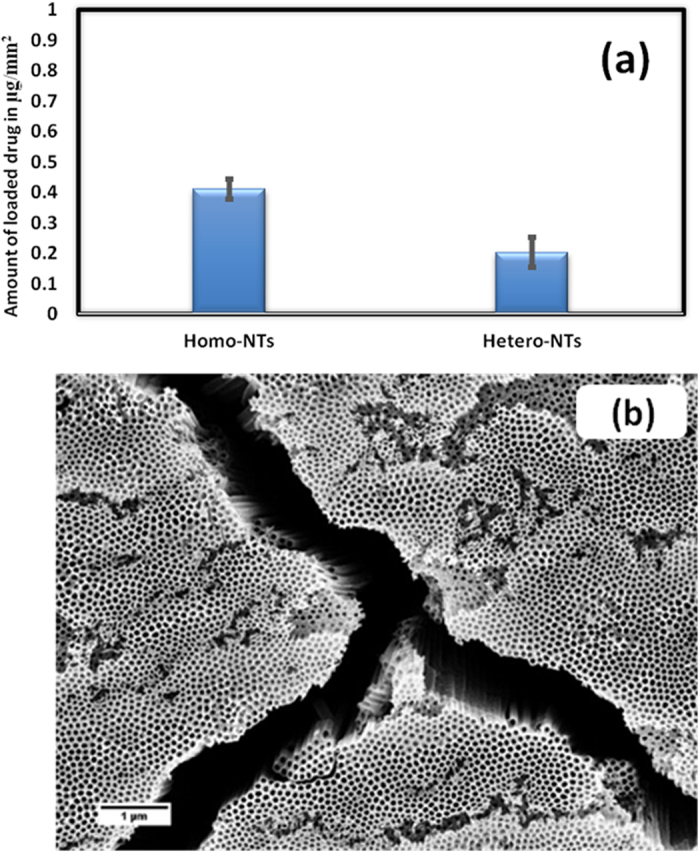
(**a**) Comparison between drug loading capacity of Homo-NTs and Hetero-NTs, (**b**) FESEM top-view image of Homo-NTs showing grooves between compact NTs islands.

The proposed platform is intended to deliver the drug only into the vascular tissue side and not the vessel lumen. Accordingly, anodized samples were tested for drug loading only on one side of the sheet covered with the NTs layer to avoid interference or duplication of results from the other side. The results were compared between the two NTs morphologies to assess the system superiority, Fig. 9a. Note that the Homo-NTs drug loading capacity is almost double that of the Hetero-NTs, which can be related to the grooves found within the Homo-NTs between islands of compact nanotubes, Fig. 9b. These grooves can reach a width of 1 μm, which can act as a potential reservoir for larger amounts of drug. As described, Homo-NTs showed more promising results for both biological response and drug loading. Accordingly, it was further studied for its drug release profile using computational analysis.

**Figure 10:**
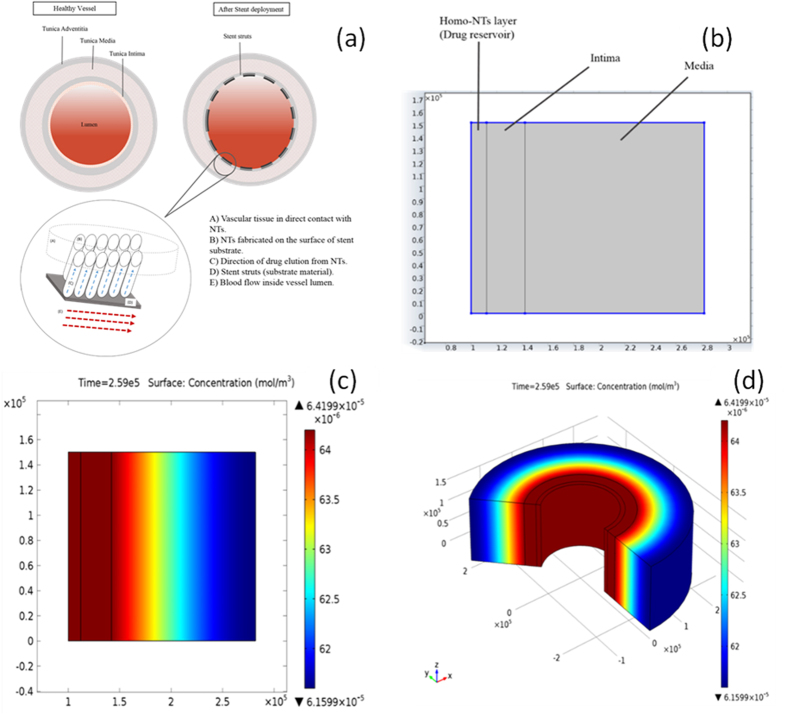
(**a**) Illustration of physical model representing the NTs drug delivery system into vessel’s tissue. The Figure is just diagrammatic representation, not representing actual relative dimensions of the system components, (**b**) 2D geometry of the modeling domains for drug release from NTs, and (**c**,**d**) Drug concentration over the modelling domain with A) 2D and B) 3D geometry at the end of simulation (3 days).

Figure 10a shows a representation of the model used for the drug release system, where cross sections of the vessel at the healthy state (on the left) and after stenting (on the right) are shown. After stenting, metal struts are embedded into the intima with direct contact with the connective tissue, while endothelium is almost damaged. Atherosclerotic plaque is hypothetically considered to be totally removed during PTCA. The Homo-NTs are grown vertically on the Ti-alloy substrate, which are embedded in the vessel wall tissue. Drug release is intended to be in the direction of vessel wall only, hence not affected by the central blood flow in the vessel lumen. Therefore, mass transport in the model was identified to be dominated by diffusion, excluding both convection and migration. Drug release was assumed to be restricted to the surrounding tissue moving across the connective tissue of Intima layer – as endothelium lining is damaged – and into the Media. Boundary conditions were set to restrict the flux within that system and to indicate that flux outside the boundaries is zero (n. Ni = 0). Accordingly, the model geometry was built as shown in Fig. 9b with 2D spatial dimension, comprising the modeling domains within the system boundaries; the NTs and Intima layer (excluding damaged endothelium and Media layer). The simulation parameters were identified reference to previously reported practical results for nanotubes diameters and same loaded drug. The materials of the model domain were identified according to their diffusion coefficient (D_c_). For Intima, D_c_ is = 5.4 × 10^−12^ m^2^/s and for Media D_c_ is = 5.0 × 10^−14^ m^2^/s^46^,^47^. This difference in D can directly affect the drug elution, as diffusion coefficient can be the rate limiting step for mass transport across the system. Diffusion coefficient of the drug in the NTs was calculated using equation (1)^48^:


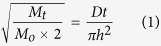


where *M*_*t*_ is the amount of drug released at time *t, M*_*o*_ is the initial drug amount, and *h* is the layer thickness. These values were retrieved from practical data measured for the same drug by Kang, *et al*.^49^. The calculated value of the drug diffusion coefficient was found to be 2.5 × 10^−11^ m^2^/s. Using the aforementioned parameters, a time-dependant study was designed for the drug release profile. Figure 10c,d shows drug concentration across the modeling domains, using the 2D geometry and its 3D representation by axial symmetry at the end of the simulation (3 days). The darkest red area represents the higher drug concentration, while the darkest blue area represents the lower concentrations.

**Figure 11:**
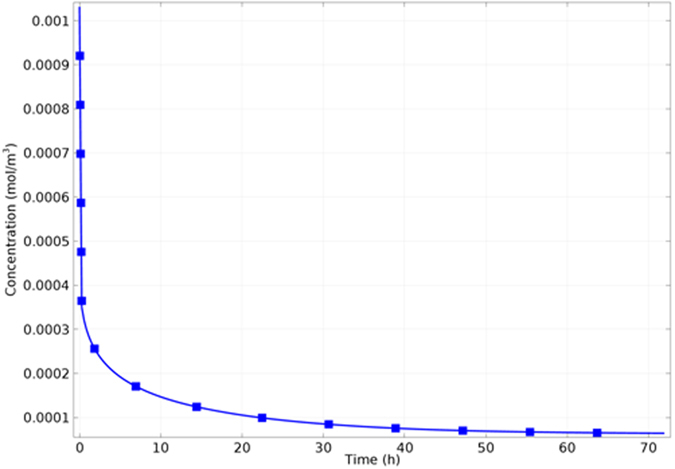
Calculated drug concentration in the NTs over time.

The time-dependant estimation of the released drug was used to plot the drug concentration in the NTs over time. As seen in Figure 11, almost 100% of the initial drug amount was released after 3 days. For DES, this rate can be considered faster than required, in reference to one month with polymeric coating. This may need more optimization of the system *via*, for example, using drug carriers or external fields^50^,^51^,^52^. However, other factors may have resulted in such relatively fast rate other than the inherent properties of the NTs system. This may include the initial amount of loaded drug. It is assumed that drug loading may increase by increasing the initially added drug and the techniques used for loading other than static solutions. This may not only increase the total amount of drug released but also the elution rate. Moreover, the nature of the drug is a critical factor in such context. The tested drug (2′-deoxyadenosine) is hydrophilic with lower diffusion coefficient than hydrophobic drugs used mainly in the market. Extending the measurement into.

## Conclusions

A biologically active, and possibly drug bearing system was proposed to replace polymeric coating on stent as a surface modification. This system comprises self-grown Ti-17Nb-6Ta-1O nanotubes that are of potential use in fabricating self-expandable stents. Two nanotubes morphologies were successfully fabricated from the same substrate using anodization technique: 1) Homo-NTs, which are highly ordered, vertically aligned nanotubes of uniform and homogeneous tube diameters, closely packed into islands separated by grooves, and 2) Hetero-NTs, which are highly ordered, vertically aligned nanotubes but of non-uniform and heterogeneous tube diameters, yet evenly distributed along the substrate. Although the XRD and Raman analyses of the as-anodized samples indicated the formation of the anatase phase associated with annealed titania NTs, the XPS analysis confirmed the formation of mixture oxides of the alloying materials on the surface. Using nanoindentation technique, Homo-NTs showed the higher hardness, while Hetero-NTs showed higher stiffness values. The MTT assay indicated that both NTs morphologies as well as the substrate material had no cytotoxicity and were ready for further biological investigation. Also, the proliferation studies showed significantly better results for endothelial cells proliferation upon using the NTs compared to their smooth counterpart. Furthermore, Homo-NTs showed superior activity than Hetero-NTs regarding biological response. Drug loading capacity were practically investigated and compared for the two morphologies using 2′-Deoxyadenosine drug, where the results were as well in favour of Homo-NTs, showing higher amount of drug retained from initially added concentration. The drug release profile for this system was then simulated and calculated through computational modeling using COMSOL Multiphysics software with transport of diluted species module. Almost 100% of the practically loaded drug was eluted from the NTs within 3 days.

## Additional Information

**How to cite this article**: Saleh, Y. E. *et al*. Functional Nanoarchitectures For Enhanced Drug Eluting Stents. *Sci. Rep.* **7**, 40291; doi: 10.1038/srep40291 (2017).

**Publisher's note:** Springer Nature remains neutral with regard to jurisdictional claims in published maps and institutional affiliations.
